# Perinatal stabilisation of infants born with congenital diaphragmatic hernia: a review of current concepts

**DOI:** 10.1136/archdischild-2019-318606

**Published:** 2020-03-13

**Authors:** Emily J J Horn-Oudshoorn, Ronny Knol, Arjan B Te Pas, Stuart B Hooper, Suzan C M Cochius-den Otter, René M H Wijnen, Thomas Schaible, Irwin K M Reiss, Philip L J DeKoninck

**Affiliations:** 1 Division of Neonatology, Department of Paediatrics, Erasmus MC University Medical Center, Rotterdam, The Netherlands; 2 Division of Neonatology, Department of Paediatrics, Leiden University Medical Center, Leiden, The Netherlands; 3 The Ritchie Centre, Hudson Institute for Medical Research, Monash University, Melbourne, Victoria, Australia; 4 Intensive Care and Department of Paediatric Surgery, Erasmus MC University Medical Center, Rotterdam, The Netherlands; 5 Department of Neonatology, University Children's Hospital Mannheim, University of Heidelberg, Mannheim, Germany; 6 Department of Obstetrics and Gynaecology, Erasmus MC University Medical Center, Rotterdam, The Netherlands

**Keywords:** congenital abnorm, resuscitation, neonatology, physiology, fetal medicine

## Abstract

Congenital diaphragmatic hernia (CDH) is associated with high mortality rates and significant pulmonary morbidity, mainly due to disrupted lung development related to herniation of abdominal organs into the chest. Pulmonary hypertension is a major contributor to both mortality and morbidity, however, treatment modalities are limited. Novel prenatal and postnatal interventions, such as fetal surgery and medical treatments, are currently under investigation. Until now, the perinatal stabilisation period immediately after birth has been relatively overlooked, although optimising support in these early stages may be vital in improving outcomes. Moreover, physiological parameters obtained from the perinatal stabilisation period could serve as early predictors of adverse outcomes, thereby facilitating both prevention and early treatment of these conditions. In this review, we focus on the perinatal stabilisation period by discussing the current delivery room guidelines in infants born with CDH, the physiological changes occurring during the fetal-to-neonatal transition in CDH, novel delivery room strategies and early predictors of adverse outcomes. The combination of improvements in the perinatal stabilisation period and early prediction of adverse outcomes may mitigate the need for specific postnatal management strategies.

## Introduction

Congenital diaphragmatic hernia (CDH) is a rare birth defect that affects around 1 in 2500 live births.^1–3^ Despite improvements in both prenatal and postnatal healthcare this condition is still associated with significant neonatal mortality, with rates varying between 30% and 50% and the majority of deaths occurring in the first year of life.^1 3^

The diaphragmatic defect can be surgically closed after birth, but the degree of disrupted lung development aggravated by herniation of abdominal organs into the chest determines survival.^4 5^ The resulting pulmonary hypoplasia is characterised by abnormal airways and vascular structures with increased smooth muscle deposition, leading to respiratory insufficiency and cardiovascular dysfunction after birth.^4 5^ The main consequence of this cardiovascular dysfunction is pulmonary hypertension (PH), which results from underdevelopment of pulmonary vessels.^6^ Treating PH in CDH infants is challenging and severe PH at the age of 1 month is associated with a mortality rate of 56% at discharge.^7 8^ The incidence of PH in the first week of life was recently reported at almost 70% in a cohort of nearly 3500 CDH infants.^9^ Treating PH—or preventing it—is accordingly a major target in CDH infants.

In recent decades, researchers have focused on developing antenatal interventions aimed at both improving pulmonary development by increasing the gas exchange area, as well as reducing PH. The benefit of temporary tracheal occlusion using fetoscopy is currently evaluated in a randomised clinical trial(Tracheal Occlusion To Accelerate Lung Growth trial, NCT00763737/NCT01240057) and the results are expected this year. In preclinical research antenatal administration of vasodilator drugs, such as sildenafil, resulted in improved pulmonary vessel development; its efficacy is being investigated in a small clinical phase I/IIb trial.^10^ The efficacy of both surfactant and antenatal steroids has been evaluated in experimental and clinical trials, but these showed conflicting results for both therapies. Indeed, the apparent benefit in experimental trials could not be replicated in a clinical setting.^11–20^ Current postnatal protocols regarding treating PH in CDH are mostly based on trials performed in infants with persistent PH of the newborn.^21^ However, infants with CDH are often excluded from these studies and in fact most of these postnatal strategies do not seem effective in treating PH in CDH, emphasising the necessity of developing novel approaches.

In this review, we will focus on the perinatal stabilisation period in the delivery room. We will summarise the available guidelines and discuss the known science on how the sequence of physiological changes is altered in infants with CDH. In addition, we will provide an overview of novel delivery room strategies that aim to improve short and long-term outcomes as well as early predictors of adverse outcomes.

## Delivery room management: current guidelines

Current guidelines regarding the resuscitation of CDH infants are predominantly opinion based, since trials assessing delivery room management are lacking. Several large European centres published a consensus document, which was updated in 2015 (CDH EURO Consortium), and a similar guideline by the Canadian CDH Collaborative was published recently.^22 23^ In contrast, the American Pediatric Surgical Association guideline does not provide recommendations regarding delivery room management in CDH infants.^24^

In both the European and Canadian guidelines, the key principle of stabilisation is to establish adequate perfusion and oxygenation while avoiding high airway pressures.^22 23^ Accordingly, most infants are intubated immediately after birth. However, in cases that are predicted to have good lung development based on prenatal assessment, a trial of spontaneous breathing could be considered according to the European guideline.^22^ The necessity of immediate support translates into immediate cord clamping (ICC) yet both guidelines do not dictate the exact timing of cord clamping.^22 23^ Moreover, for all recommendations the level of evidence was graded as very low. This emphasises the necessity of generating robust scientific evidence that supports these treatment strategies.

## Transition period

### Fetal phenotype

**Figure 1:**
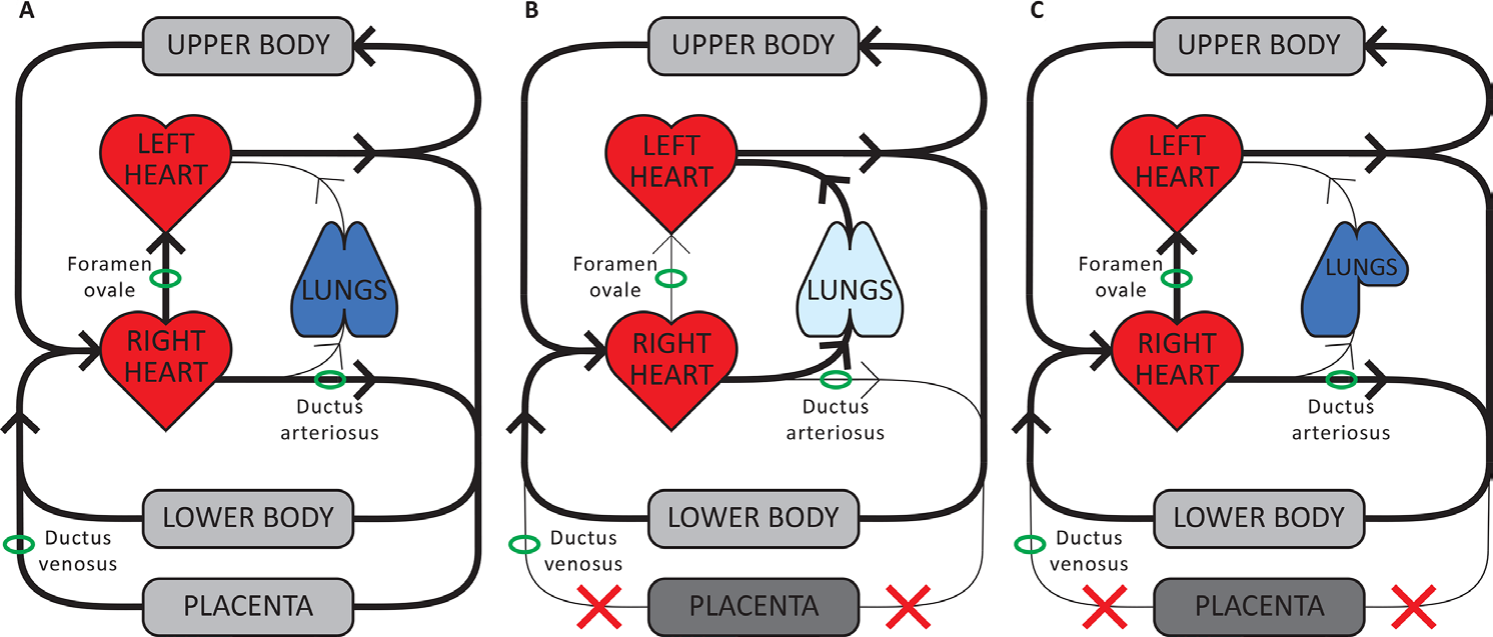
Cardiopulmonary physiology. (A) Fetal phenotype: left ventricular (LV) preload is mainly derived from umbilical venous blood which flows via the ductus venosus and foramen ovale directly into the left atrium. As pulmonary vascular resistance (PVR) is high, pulmonary blood flow (PBF) is low and the majority of right ventricular output bypasses the lungs and flows through the ductus arteriosus into the descending aorta. A high proportion of cardiac output flows through the low-resistance placental vascular bed, which contributes to a low-systemic vascular resistance (afterload). (B) Fetal-to-neonatal transition in healthy infants: umbilical cord clamping decreases LV preload and increases systemic vascular resistance by removing both umbilical venous return and the low-resistance placental vascular bed. Lung aeration triggers a decrease in PVR and an increase in PBF, which then re-establishes LV preload lost upon cord clamping. The lungs then take over both the cardiovascular and respiratory functions of the placenta. (C) Fetal-to-neonatal transition in infants with congenital diaphragmatic hernia: as airway liquid clearance is delayed, the decrease in PVR and increase in PBF are also delayed. As such, clamping of the umbilical cord immediately after birth results in a prolonged period of reduced cardiac output due to a loss in preload. As cardiac output is critical for defending the infant against hypoxia, a prolonged period of low cardiac output combined with limited or no oxygen exchange greatly increases the risk of hypoxic/ischaemic brain injury.

In the fetal circulation (figure 1A), left ventricular (LV) preload is mainly derived from umbilical venous blood flow that is preferentially streamed to the left atrium via the ductus venosus and the right-to-left shunt through the foramen ovale.^25 26^ The placental circulation provides oxygenation of fetal blood, and greatly reduces overall systemic vascular resistance because placental vascular resistance is low.^25 26^ Pulmonary vascular resistance (PVR) is high since the fetal lungs are not aerated and as a result pulmonary blood flow (PBF) is low. Thus, the majority of the right ventricular output shunts from right to left through the ductus arteriosus to enter the systemic circulation.^25^

### Transition to neonatal phenotype in healthy newborns

At the time of cord clamping several adaptations in the cardiovascular and pulmonary system occur as the low resistance placental vascular bed is removed from the systemic circulation (figure 1B). The loss of umbilical venous return decreases LV preload by 30%–50% and, when combined with an instantaneous increase in systemic vascular resistance, cord clamping results in a decrease in cardiac output as reflected by lower stroke volume and heart rate.^25 26^ This likely explains the observed transient bradycardia in the heart rate nomograms of normal healthy infants.^27 28^ Lung aeration triggers the transformation of the fetal circulation into the neonatal phenotype by stimulating a decrease in PVR and an increase in PBF, which then re-establishes the preload lost on cord clamping.^25^ In healthy newborns, lung aeration is established rapidly after birth thereby avoiding severe hypoxia.^5^

### Transition to neonatal phenotype in CDH

For almost all infants born with CDH, transition to the neonatal phenotype is complicated and support from medical professionals is essential for survival. However, our knowledge of how pulmonary hypoplasia affects the cardiopulmonary physiological changes during the transition period is largely based on animal models.

Hypoplastic lungs have low compliance, resulting in delayed lung aeration after birth (figure 1C).^29^ In particular, airway liquid clearance will be impeded because of an increased resistance to liquid flow down through the airways due to their smaller total cross-sectional area, in combination with a reduced surface area for liquid movement into the perialveolar tissue.^29^ Since lung aeration is the most important trigger for decreasing PVR and augmenting PBF, impaired airway liquid clearance and thus delayed lung aeration results in a delay in the sequence of physiological changes that transform the fetal circulation into a neonatal circuit.^5 25 30^ Accordingly, after cord clamping there is a prolonged period of reduced cardiac output, resulting in gradually worsening of hypoxia and blood pressure fluctuations in the pulmonary and systemic vasculature.

Postnatal LV dysfunction—both systolic and diastolic—adds to the haemodynamic instability during transition in these CDH infants.^31 32^ The aetiology of LV dysfunction in CDH is multifactorial and is related to the relatively small size, resulting from both the herniation of abdominal organs as well as the altered LV filling haemodynamics.^32 33^ After cord clamping, LV dysfunction most likely results from reduced LV filling due to the limited increase in PBF (eg, preload), LV hypoplasia and acute increase in afterload. Moreover, there is transmission of right ventricular dysfunction to the LV via ventricular interdependence.^31 32^

In an ovine diaphragmatic hernia (DH) model, the lambs developed respiratory acidosis and poor cerebral oxygenation immediately after birth, confirming the hypothesised worsening of hypoxia after ICC.^30^ Hypoxia may result in an enhanced vasoconstrictive response since the pulmonary vasculature in CDH infants is hyper-reactive.^30^ The resulting increase in vascular resistance could worsen PH, ultimately leading to an increase in the pre-existing right-to-left shunt, which in turn would aggravate the hypoxia and trigger an ongoing cycle of worsening PH.^5 8 30 34^

## Delivery room management: novel concepts

Improving outcomes of infants born with CDH requires clinical trials assessing innovative strategies in these infants. Considering it is a rare condition, large trials may not always be feasible as recruitment is not sufficient to achieve statistical and clinical significance within a reasonable time frame. This is also reflected by the fact that all concepts described in this paragraph have a low grade of recommendation (D). We will discuss early predictors of adverse outcomes and innovative treatment strategies during the perinatal stabilisation period that seem promising based on preclinical studies.

### Early prediction of adverse outcomes

Several early prediction models and parameters during the immediate postnatal period have been suggested, which could allow initiation of prophylactic therapies in an attempt to individualise healthcare for each neonate. Herein we will discuss two parameters and three prediction models.

#### Tidal volume

In a prospective study, tidal volume (Vt) of spontaneous breaths during stabilisation was associated with mortality, chronic lung disease incidence and the need for inhaled nitric oxide.^35^ However, Vt was correlated with the observed to expected lung-to-head ratio (o/e LHR) and was therefore not considered an independent predictor.^35^ Still, it may be useful in confirming disease severity at birth.^35^

#### Oxygenation index

The oxygenation index (OI), a value calculated to estimate the severity of respiratory failure, may predict mortality in CDH infants.^36 37^ The highest OI recorded in the first 48 hours was positively associated with higher mortality, greater length of stay and more ventilator days in a retrospective study.^37^ A subsequent retrospective study confirmed that reported mean and best OI on day 1 were reliable predictors for mortality.^36^ Calculating the OI requires the arterial PO_2_ value, which is obtained by means of arterial blood gas sampling. However, as most CDH infants have a patent ductus arteriosus at this time and preductal PaO_2_ values are thus higher than postductal PaO_2_ values, the site of blood gas sampling could result in a major difference in the calculated OI.^31^ Moreover, arterial blood gas sampling is invasive, requiring an arterial line or intermittent arterial puncture.^38^ The use of the oxygenation saturation index, a non-invasive measure, can potentially alleviate these disadvantages of OI, although its use and usefulness in CDH infants has not been established.^38^

#### Score for Neonatal Acute Physiology-II

The Score for Neonatal Acute Physiology-II (SNAP-II Score) was first created in 2001 and is calculated with the use of arterial blood pressure, pH, PaO_2_:FiO_2_ ratio, body temperature, diuresis and seizure activity.^39^ A prospective trial assessing the initial ventilation strategy in CDH infants showed the predictive value of SNAP-II for both mortality as well as the need for extracorporeal membrane oxygenation (ECMO).^40^ The SNAP-II Score is easy to use and its potential as early predictor was also confirmed in a more recent retrospective study.^41^

#### Wilford Hall/Santa Rosa prediction model

The Wilford Hall/Santa Rosa prediction formula was developed as an easy-to-use clinical method for predicting outcomes in the first 24 hours after birth.^42^ This formula is calculated by PaO_2_[max]−PaCO_2_[max] and the authors hypothesised that in CDH infants with severe PH, arterial PO_2_ would be less than PCO_2_ as result of compromised gas exchange. However, the clinical accuracy of this model was not sufficient in predicting outcomes of individual patients, and a subsequent clinical trial reported poor discrimination in predicting outcomes as well.^42 43^

#### Brindle scoring model

Brindle *et al* developed a third clinical prediction model in 2014.^44^ This model provides a simple and generalisable scoring system discriminating between low (<10%), intermediate (~20%) and high (~50%) risks of mortality.^44^ This CDH scoring equation includes five predictors: 5 min Apgar, birth weight, PH, chromosomal anomalies and major cardiac defects.^44^ External validation has confirmed good performance and discrimination among the three groups.^45^

### Ex utero intrapartum treatment-to-ECMO procedure

Many of the severely affected CDH infants are treated with ECMO, although its efficacy in CDH remains debatable. The apparent small gain in survival rates may be offset by increased long-term disability in survivors.^46^ A potential explanation for this increased morbidity is that ECMO treatment is usually only considered when infants are already critically ill. In an attempt to improve the benefits of ECMO, the ex utero intrapartum treatment-to-ECMO (EXIT-to-ECMO) procedure was suggested for the patients with most severe CDH (<15% predicted lung volume).^47 48^ The first report demonstrated no clear survival benefit of EXIT-to-ECMO: mortality was higher than in the non-EXIT group.^48^ On the other hand, there was a higher incidence of cardiac anomalies in the EXIT-to-ECMO group.^48^ A second pilot series by the same group evaluated the potential benefit on morbidity outcomes.^47^ While the limited sample size (eight EXIT-to-ECMO vs nine non-EXIT) precludes firm conclusions from being drawn, there are some indications suggesting that survivors of the EXIT-to-ECMO treatment were healthier.^47^ The authors, however, conclude that based on their experience, which is the largest to date, the use of EXIT-to-ECMO for severe CDH infants has not resulted in improved outcomes.^47 48^

### Initial respiratory support

Respiratory support is a major topic in delivery room management for CDH infants, however, it is a double-edged sword as the importance of ventilator-induced lung injury (VILI) and oxygen toxicity is not to be underestimated. ‘Gentle ventilation strategies’ and permissive hypercapnia have been implemented in delivery room management and on intensive care units, as less aggressive ventilator strategies could reduce the risk of VILI and oxygen toxicity.^22^ However, the optimal way of guiding oxygen supplementation in CDH infants is still not clear. The possibility of causing inadvertent cerebral hyperoxia was highlighted in a recent study using an ovine DH model.^5^ The authors hypothesised that this occurs when the FiO_2_ levels are rapidly increased in response to reduced SpO_2_ levels without knowledge of cerebral blood flows, leading to a rapid increase in cerebral oxygen delivery.^5^ To directly assess cerebral oxygenation, they have speculated that ventilation management may be better guided by the use of near-infrared spectroscopy.^5^

The CDH EURO Consortium also recommends that CDH infants may benefit from starting with FiO_2_ lower than 1.0.^22^ While there is minimal evidence to support this recommendation, reduced generation of free radicals associated with lower FiO_2_ levels may prevent subsequent pulmonary vasoconstriction.^49^ In a retrospective cohort series that compared starting resuscitation of CDH infants with FiO_2_ 0.5 vs 1.0 (68 vs 45 patients), no differences in immediate postnatal outcomes were found.^50^ However, infants who required an increase in FiO_2_ during stabilisation were more likely to have an adverse outcome.^50^

In infants predicted to have mild hypoplasia that are potentially capable of a smooth transition into neonatal life, the initiation of invasive respiratory support may actually cause damage to the lungs. To avoid VILI for this specific group—infants with a left-sided defect, intra-abdominal liver and o/e LHR ≥50%, a trial of spontaneous breathing was postulated in the consensus CDH EURO Consortium guideline.^51^ The benefit of such an approach has not been determined objectively, yet this has been explored in the Erasmus MC and a manuscript reporting our experience over a 5-year period is currently under review.

### Timing of cord clamping

The importance of appropriate timing of cord clamping in optimising perinatal stabilisation of newborns has been evaluated comprehensively over the last decade.^2 52 53^ Delayed cord clamping (DCC) can be performed using either a ‘time-based’ or a ‘physiological-based’ approach, with the latter focusing on the infant and its physiological stability rather than a stopwatch. Indeed, the high variability between newborns in their needs and outcomes already suggests that DCC at an arbitrarily chosen point of time may not be sufficient.^25^ Initially, the benefits of DCC were attributed to an increase in placental transfusion, reducing the incidence of neonatal anaemia in both term and preterm infants. In preterm infants, however, the benefit of DCC may be more related to establishing lung aeration prior to cord clamping, resulting in a smooth cardiopulmonary adaptation at birth.^26^

In physiological-based cord clamping (PBCC), the umbilical cord is clamped after the infant has established lung aeration with spontaneous breathing or respiratory support.^53 54^ Lung aeration before cord clamping allows the pulmonary vasculature to dilate and PBF to increase, while maintaining LV preload through umbilical venous return.^54 55^ Thus, an elevated PBF can immediately take over the maintenance of LV preload as soon as the cord is clamped, allowing the preservation of LV filling and function throughout the transitionary period.^31^ This would lead to a better cardiopulmonary transition compared with ICC, with prevention of hypoxia and loss of LV output. This concept has recently been confirmed in an ovine model, in which cardiopulmonary transition in DH lambs was compared between ICC and PBCC.^5 30^ In this experiment, PBCC translated into an increase in PaO_2_ and preductal SaO_2_, an almost 20-fold greater PBF at the time of cord clamping (p<0.001), and a significantly lower pulmonary artery pressure at 10 min after ventilation onset (p<0.001).^30^ PBCC in patients with CDH could also offer the opportunity of commencing resuscitation with 21%–50% instead of 100% of oxygen since the infants are still on the placental circulation and therefore require less supplemental oxygen.^49 56^

As mentioned before, due to the hyper-reactive pulmonary vasculature in CDH infants temporary PH could lead to a cascade ultimately resulting in persistent PH.^5 8 30 34^ Incorporating PBCC into the stabilisation of CDH infants could theoretically reduce the risk of ongoing PH in these infants simply by incorporating a low-risk intervention. If proven effective in humans, this could lead to a significant shift in delivery room strategies. The feasibility of intact cord resuscitation in CDH infants was recently demonstrated in two single-centre pilot studies; both studies did not observe maternal or neonatal adverse events with DCC.^2 57^ These trials were not designed to detect the impact of the intervention on the cardiopulmonary outcomes, although it was suggested that DCC improved early cardiorespiratory adaptation at birth.^2^ However, Foglia *et al* performed cord clamping after colorimetric carbon dioxide detection and as such, it is likely that cord clamping was performed without the infants being ‘stabilised’.^57^

Based on the promising preliminary results we will initiate a multicentre and international randomised controlled trial evaluating PBCC in CDH infants, the PinC trial (NL69575.078.19), starting in 2020. Left-sided CDH infants will be randomised to either standard approach (ie, ICC) or PBCC, meaning the umbilical cord will not be clamped until after the infant is stabilised. A consistent definition for physiological stability has not been established yet, but the goal is to assess adequate lung aeration. We decided to use physiological parameters as heart rate and oxygenation as proxy for aeration, because of its simplicity and is analogous to what is currently used in a trial evaluating the outcomes of PBCC in very preterm neonates.^58^ The PinC trial is designed to detect the impact of PBCC on clinical outcomes, for example, the occurrence of PH diagnosed in the first 24 hours after birth. In parallel, there are other ongoing and anticipated initiatives evaluating intact cord resuscitation in CDH infants in Denver (CO, USA) and France (nationwide), respectively.

## Summary

Infants with CDH will face important respiratory challenges immediately after birth and in some patients, deterioration of their clinical situation may be the result of our current management strategies at birth. We propose that this earliest time period of newborn life has the potential to provide both reliable markers for prediction of outcomes, such as PH, and the possibility to adopt innovative management strategies in an attempt to have more children survive. Optimising perinatal stabilisation could protect hypoplastic lungs from damage due to the treatments that we employ to save their lives.
